# Preparation of fast-swelling porous superabsorbent hydrogels with high saline water absorbency under pressure by foaming and post surface crosslinking

**DOI:** 10.1038/s41598-023-40563-1

**Published:** 2023-08-24

**Authors:** Naihua Zhai, Baogui Wang

**Affiliations:** College of Chemistry and Pharmaceutical Sciences, Qingdao Agricultural University, Qingdao, 266109 People’s Republic of China

**Keywords:** Materials science, Soft materials

## Abstract

Superabsorbent hydrogels have wide applications in many fields because of their unique water absorbing performance. In spite of decades of research about superabsorbent hydrogels, high water absorbency under pressure and fast-swelling are still challenging and highly desired for their applications in hygienic products and others. Here, we report preparation of fast-swelling porous starch-*g*-poly(acrylic acid)/poly(vinyl alcohol) superabsorbent hydrogels with high saline water absorbency under pressure by foaming and post surface crosslinking. 2,2′-Azobis(2-amidinopropane) dihydrochloride (AIBA) was used as a new porogen instead of conventional porogens like NaHCO_3_. Post surface crosslinking of the hydrogel was achieved using glycerol via the esterification reaction. AIBA is a better porogen than NaHCO_3_ regarding porosity and swelling performance of the hydrogels, and its content has great influences on structure and swelling performance of the hydrogels including water absorbency and swelling rate. Also, the surface crosslinking using glycerol can significantly enhance the saline water absorbency under pressure (2 kPa) but at the sacrifice of the swelling rate. Consequently, the hydrogels show high water absorbencies for deionized water (560 g/g), 0.9 wt% NaCl solution (58 g/g), 0.9 wt% NaCl solution under 2 kPa pressure (28 g/g) and fast-swelling (31 s to achieve a highly swelling state).

## Introduction

Since the first paper about hydrogels reported by Wichterle and Lim^1^, hydrogels have been widely used in chemical engineering^2–4^, agricultural^5–7^, pharmaceutical^8,9^, architecture^10,11^, and hygienic applications^12,13^, etc. For the applications in hygienic products, one of the very important properties is fast swelling of dry hydrogels in aqueous solutions. Generally, the rate of penetration of water molecules into the polymeric network and the hydrophilicity of the functional groups are the two major factors determining the swelling rate of hydrogels^14,15^. Thus, higher porosity in the hydrogels can increase the contact area between the polymeric network and external aqueous solutions, which is helpful to enhance the swelling rate^16^.

Until now, various pore-forming methods including the phase-separation technique^17^, freeze-drying and hydration technique^18^, the porosigen technique^19^, and the foaming technique^20,21^ have been employed to prepare porous hydrogels. Among these methods, the foaming technique is a simple and effective method for generating porous structure in hydrogels. Carbonate compounds such as NaHCO_3_^22^ and organic solvents such as methanol and acetone are the most frequently used foaming reagents^23,24^. In addition, porous hydrogels obtained through foaming in the polymerization process and non-solvent dewatering of the as-synthesized hydrogels were also reported^25^. Even though the porosity and the swelling rate of hydrogels can be improved by using carbonate compounds as porogens, however, the time and sequence of adding porogens as well as the gelation time of the polymerization process need to be carefully controlled because the acid-induced decomposition of carbonate compounds occurred intensively upon adding them into the reaction mixture. In addition, it is difficult to obtain a hydrogel with homogeneous porous structure due to the nonuniform dispersion of carbonate particles in the reaction mixture. Hence, to find a porogen that can be well dispersed in the reaction mixture but cannot be decomposed before polymerization is necessary to obtain a stable foaming process and thus to have good porous structure.

On the other hand, for the applications in hygienic products, the water absorbency of hydrogels under pressure is important^26^. For some conventional hydrogels, the granular size of hydrogels during swelling in aqueous saline solutions may be reduced under pressure. This will greatly hinder permeability of aqueous liquids such as body fluids through the hydrogel particles and thus water absorbency of the hydrogels will be greatly reduced. It is greatly desired to have high water absorbency under pressure to meet the requirement for applications in the hygienic area. Surface crosslinking after preparation of hydrogels is a plausible approach to solve the issue without scarifying too much of the water absorbency^27,28^.

Starch-based hydrogels have attracted great attention due to their excellent biocompatibility and biodegradability^29–31^. Poly(vinyl alcohol) (PVA) is a nonionic hydrophilic polyhydroxy linear polymer, which has excellent water solubility, nontoxicity, biodegradability, compatibility and high affinity to various hydrophilic polymers^32,33^. It had been proved that introducing PVA into hydrogels could enhance their hydrophilicity, gel strength, and other properties^34–36^. However, the swelling rate of hydrogels should be further enhanced to meet the demand of practical applications.

Here, we report preparation of fast-swelling porous hydrogels with high saline water absorbency under pressure by foaming and post surface crosslinking. During preparation of the starch-*g*-poly(acrylic acid)/poly(vinyl alcohol) (St-*g*-PAA/PVA) hydrogels, 2,2′-azobis(2-amidinopropane) dihydrochloride (AIBA) or NaHCO_3_ (for comparison) was introduced as the porogen. After preparation of the St-*g*-PAA/PVA hydrogels, the dry gels were further surface-crosslinked using glycerol via the esterification reaction. The St-*g*-PAA/PVA hydrogels show high porosity, fast swelling in saline water and high water absorbency under pressure.

## Results

### Design and preparation of porous St-*g*-PAA/PVA hydrogels

**Figure 1:**
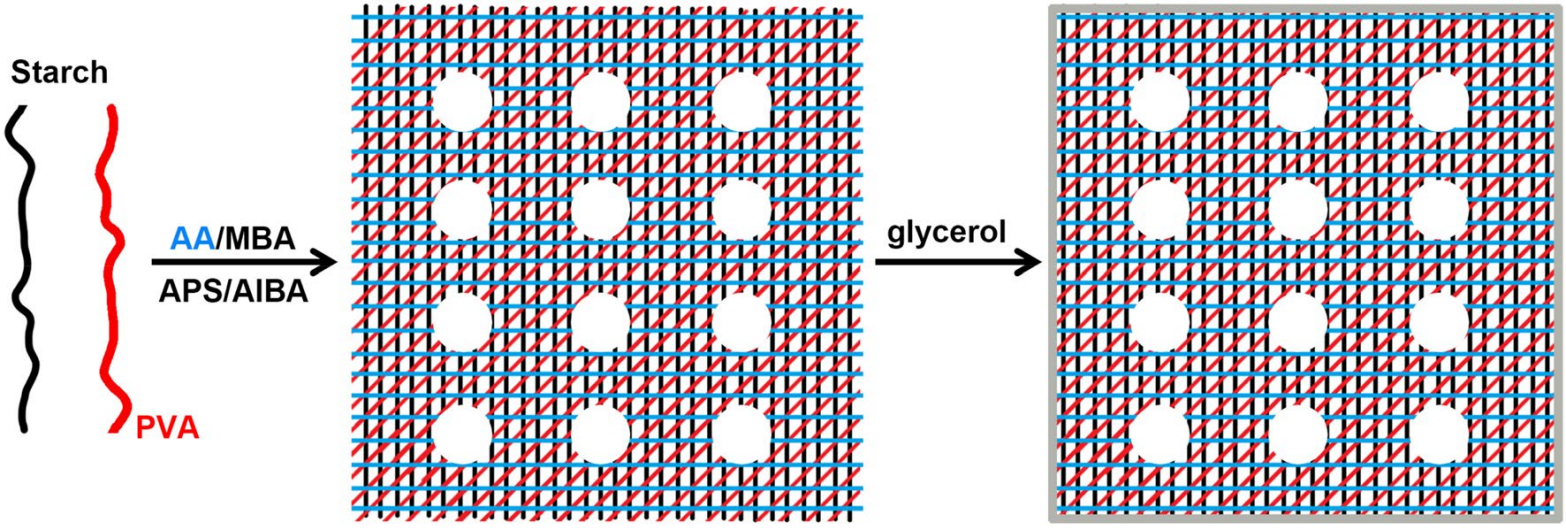
Schematic preparation of the porous St-*g*-PAA/PVA hydrogels by using AIBA as the foaming reagent and glycerol as the post surface crosslinker. Ammonium persulfate (APS) was used as the initiator.

To enhance the swelling rate, porous structure was designed in the St-*g*-PAA/PVA hydrogels by introducing a pore-forming reagent. To enhance the water absorbency under pressure, surface crosslinking of the St-*g*-PAA/PVA gels after polymerization was carried out. According to the Flory theory^37^, surface crosslinking can enhance the crosslinking density on the surface of the superabsorbent hydrogels and thus improve water absorption under pressure. Surface crosslinking is a widely used method for enhancing water absorbency under pressure^38^. Figure 1 shows preparation of the porous St-*g*-PAA/PVA hydrogels by using AIBA as the foaming reagent and glycerol as the post surface crosslinker. The St-*g*-PAA/PVA hydrogels were prepared by free radical graft polymerization of partly neutralized AA (60%) onto starch and meanwhile crosslinking with *N,N'*-methylenebisacrylamide (MBA) as the crosslinker in the presence of PVA, a hydrophilic polyhydroxy linear polymer with many advantages as mentioned in the Introduction section. Thus, a semi-interpenetrating network was form. During polymerization, AIBA was used as the foaming reagent. AIBA is a water-soluble azo initiator and can be dissociated into cationic free radicals and N_2_ at temperature above 60 °C. Also, it can react with AA to form acrylate in the reaction system, and thus disperse homogeneously in aqueous solutions without using any surfactants or water-soluble polymers. Moreover, no settlement, separation and floating phenomena were observed during the polymerization process. Therefore, it is reasonable to infer that the AIBA-based foaming process can continually and homogeneously occur in the whole reaction process. After polymerization, the St-*g*-PAA/PVA hydrogels were further surface crosslinked by spraying the glycerol crosslinker onto them followed by heat treatment at high temperature. Glycerol could react with -COOH of superabsorbent hydrogels via the esterification reaction^38^. Here, glycerol could react with the abundant -COOH groups on the surface of the St-*g*-PAA/PVA hydrogels, which could enhance surface crosslinking density of the hydrogels.

### Effects of NaHCO_3_ on structure and swelling performance of St-*g*-PAA/PVA hydrogels

**Figure 2:**
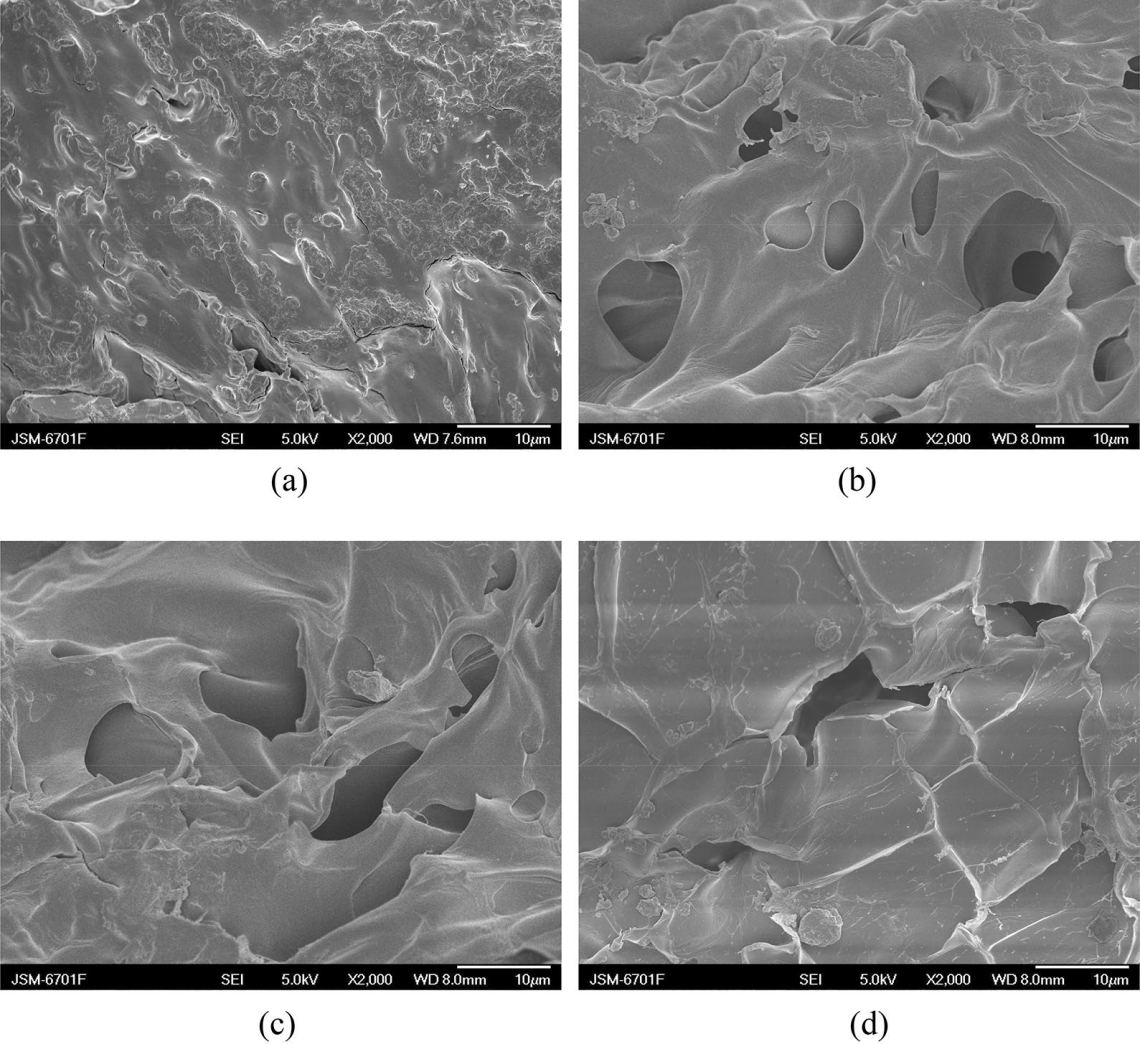
SEM micrographs of the St-*g*-PAA/PVA hydrogels with (**a**) 0%, (**b**) 3%, (**c**) 5% and (**d**) 7% NaHCO_3_.

The effect of NaHCO_3_ on structure of the St-*g*-PAA/PVA hydrogels was investigated by SEM (Fig. 2). As can be seen from Fig. 2a, the porogen-free sample shows a dense surface and no pores can be seen. In contrast, a porous structure is clearly observed for the hydrogel prepared with 3.0 wt% NaHCO_3_ (Fig. 2b). The density of the hydrogel with 3.0 wt% NaHCO_3_ is 0.93 g/cm^3^ as measured by the pycnometric method according to the ASTM D792 standard^25^. The further increase in the NaHCO_3_ content to 7.0 wt% has no obvious influence on morphology of the hydrogels (Fig. 2c,d). Note that when NaHCO_3_ was used as the porogen, it is difficult to disperse the NaHCO_3_ particles uniformly in the reaction system^25^. Moreover, the NaHCO_3_ species could react with the –COOH groups of AA immediately after adding them into the reaction system and a lot of CO_2_ bubbles will be generated, which could easily emit from the reaction system before the gelation point. Therefore, more amount of NaHCO_3_ is needed in the foaming process and most of them are useless. Also, the pore size in the hydrogels is very inhomogeneous and some pores are very large.

**Figure 3:**
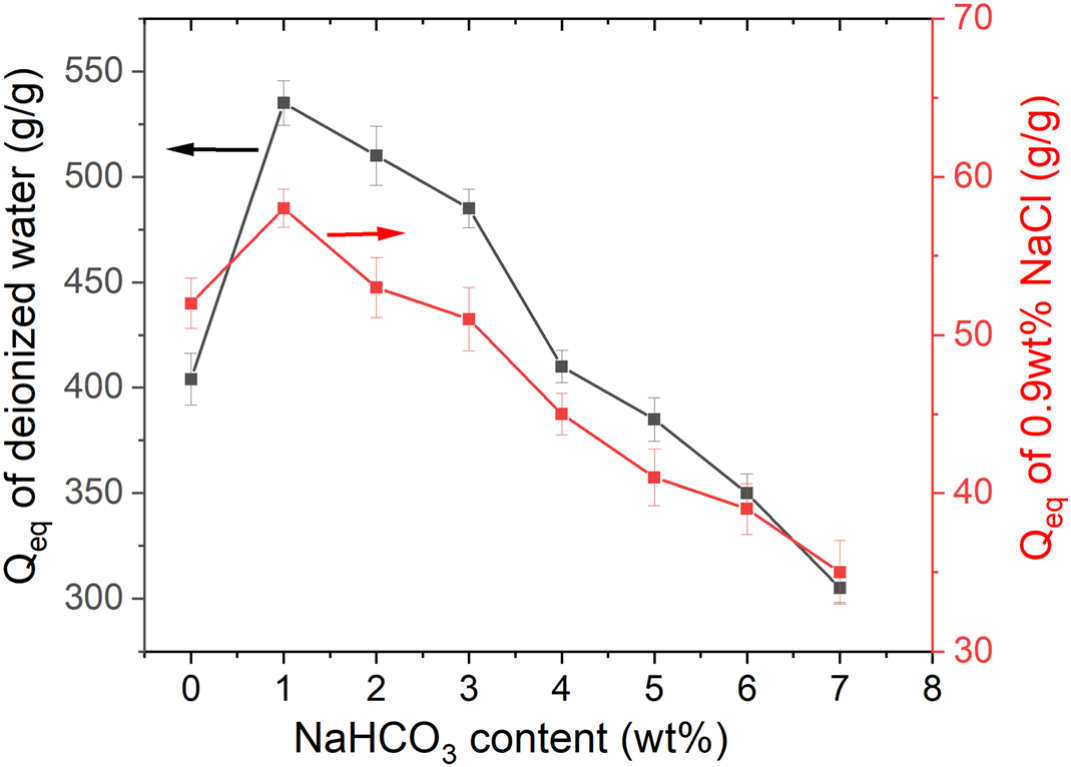
Effect of NaHCO_3_ content on water absorbency of the St-*g*-PAA/PVA hydrogels. Data are shown as mean ± SD, n = 3.

The NaHCO_3_ content also affects water absorbency of the St-*g*-PAA/PVA hydrogels (Fig. 3). The water absorbency of the hydrogel prepared without addition of porogen is 404 g/g in deionized water and 52 g/g in 0.9 wt% NaCl solution. The water absorbency was tested under atmospheric pressure unless otherwise specified. The water absorbency of the hydrogels increases to 535 g/g (deionized water) and 58 g/g (0.9 wt% NaCl) when 1.0 wt% NaHCO_3_ was used as the foaming reagent. However, the further increase in the NaHCO_3_ content to 7.0 wt% results in gradual decrease in the water absorbency to 305 g/g for deionized water and 35 g/g for 0.9 wt% NaCl solution. This is because too much NaHCO_3_ has changed neutralization degree of AA, and thus resulted in decline in the water absorbency.

**Figure 4:**
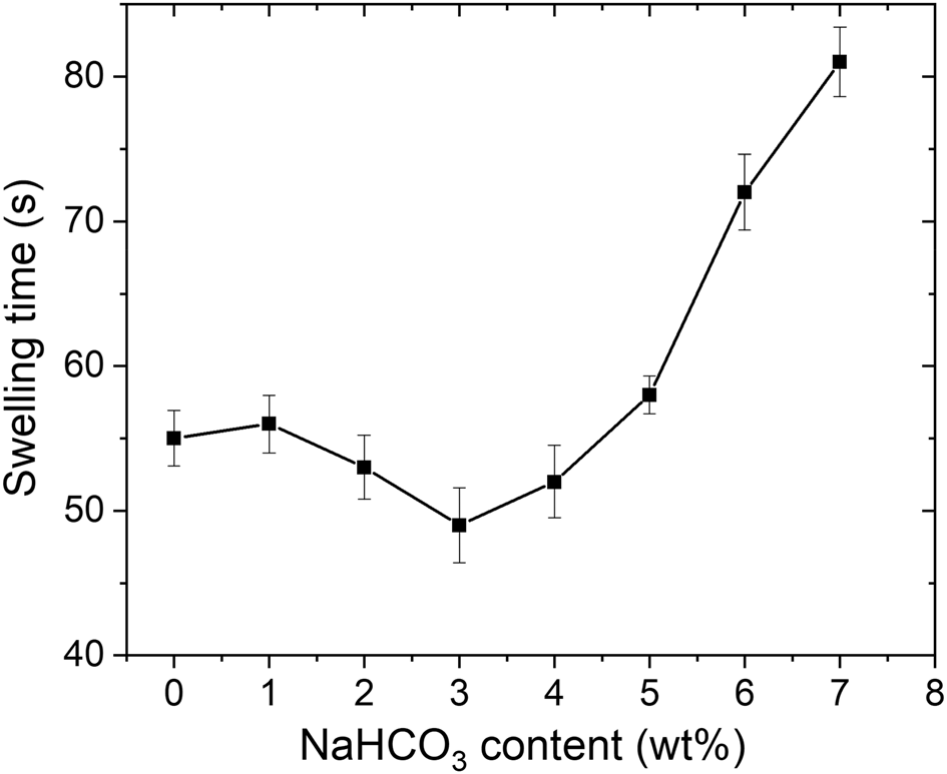
Effect of NaHCO_3_ content on swelling time of the St-*g*-PAA/PVA hydrogels. Data are shown as mean ± SD, n = 3.

The NaHCO_3_ content also affects swelling rate of the hydrogels (Fig. 4). For the hydrogel prepared without NaHCO_3_, the swirl disappeared after a swelling time of 55 s. In contrast, the swelling time was reduced to 49 s after introducing 3.0 wt% NaHCO_3_, which is mainly due to the porous structure generated by NaHCO_3_. The porous structure promotes penetration of water molecules into the hydrogel network and thus enhances the swelling rate. With further increasing the NaHCO_3_ content to 7.0 wt%, the swelling rate decreased evidently and a swelling time of 81 s was needed for the swirl to disappear. This is because of decline in the water absorbency with increasing the NaHCO_3_ content. Note that swelling rate and water absorbency do not necessarily have the same trend.

### Effects of AIBA on structure and swelling performance of St-*g*-PAA/PVA hydrogels

**Figure 5:**
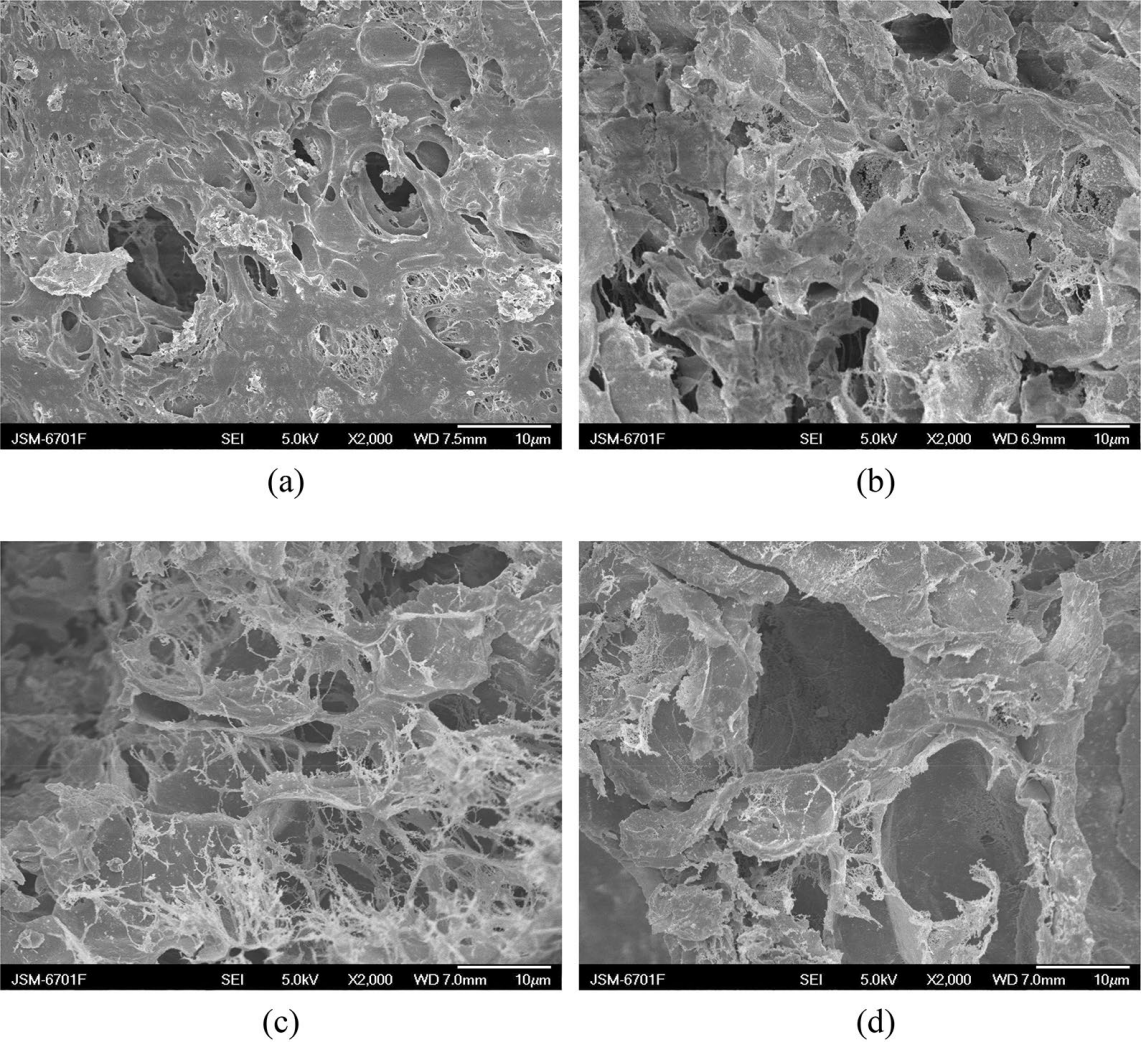
SEM micrographs of the St-*g*-PAA/PVA hydrogels with (**a**) 0.1, (**b**) 0.3, (**c**) 0.7 and (**d**) 1.1 wt% AIBA.

The effect of AIBA content on structure of the St-*g*-PAA/PVA hydrogels was also studied and compared with those prepared with NaHCO_3_ as the porogen. Different from the porogen-free and NaHCO_3_-based hydrogels, the hydrogels prepared using AIBA as the porogen are highly porous, and the pores are smaller and more uniform (Fig. 5). Also, the density of the hydrogel prepared with 0.1 wt% is merely 0.67 g/cm^3^, which greatly lower than that prepared with 3 wt% NaHCO_3_ (0.93 g/cm^3^). These results mean that the water-soluble azo initiator AIBA is a good porogen and the foaming can occur continually and homogeneously in the reaction system.

Figure 5 shows the effect of the AIBA content on the porous structure of the hydrogels. A relatively compact surface and a small number of pores are observed for the hydrogel prepared with 0.1 wt% AIBA. Whereas the surface becomes obviously more porous and some interconnected pores can be found with increasing the AIBA content to 0.3 wt%. This structure should be helpful for enhancing the swelling rate by facilitating water penetration and transport. With further increasing the AIBA content to 0.7 wt% and 1.1 wt%, many N_2_ bubbles were released during the synthesis process. Too much gas could lead to cavitation, warp and collapse, or even cracking of the hydrogels (Fig. 5c,d).

**Figure 6:**
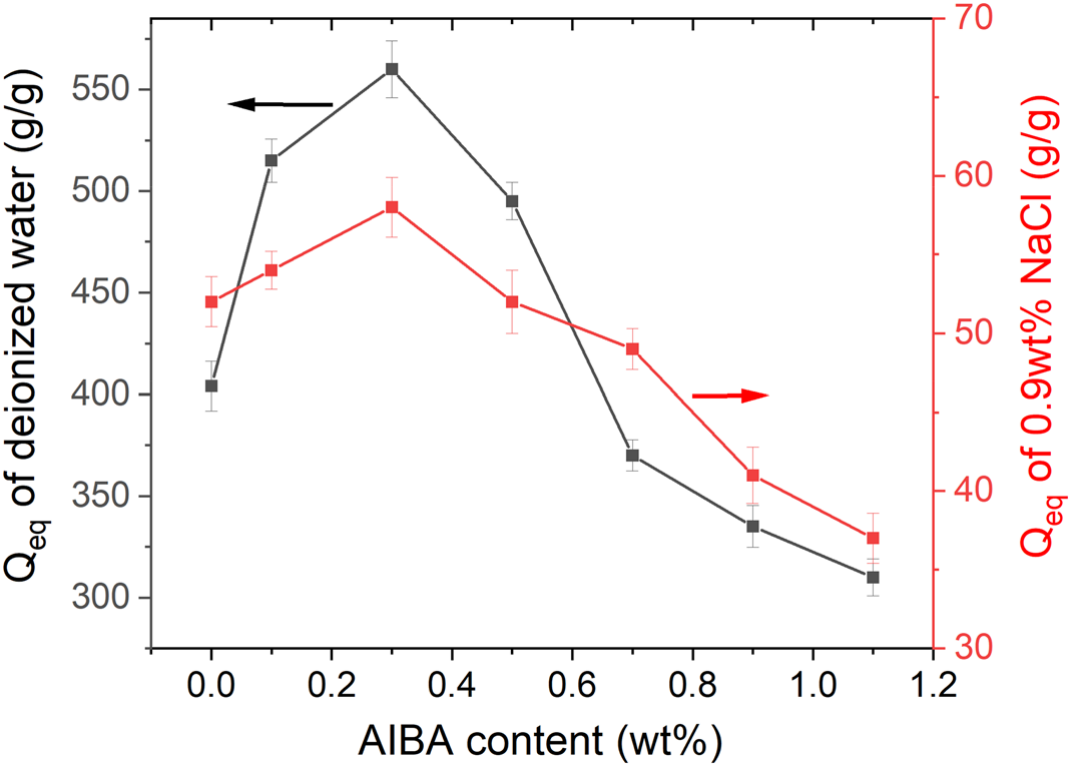
Effect of AIBA content on water absorbency of the St-*g*-PAA/PVA hydrogels. Data are shown as mean ± SD, n = 3.

The effect of the AIBA content on water absorbency of the St-*g*-PAA/PVA hydrogels is shown in Fig. 6. The changing tendency of water absorbency with the AIBA content is similar to that with the NaHCO_3_ content. The water absorbency increased with increasing the AIBA content to the maximum value of 560 g/g in deionized water and 58 g/g in 0.9 wt% NaCl solution at 0.3 wt% of AIBA. The water absorbency in deionized water is slightly higher than the hydrogel prepared with 1.0 wt% Na_2_CO_3_. Then, the water absorbency decreased with further increasing the AIBA content.

**Figure 7:**
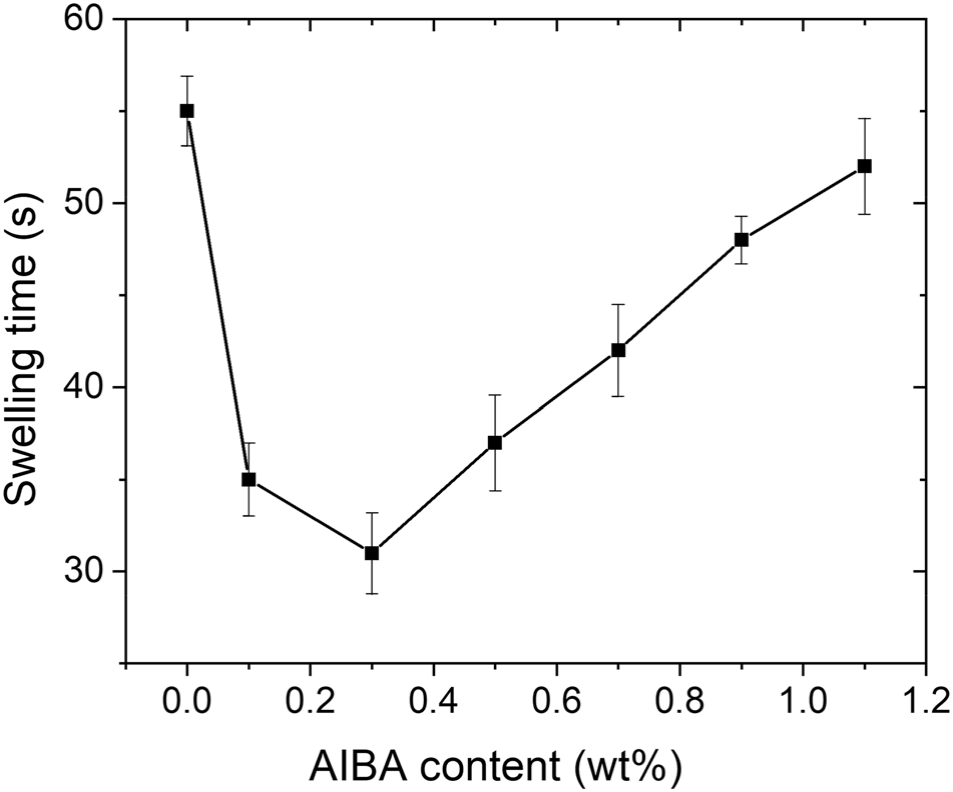
Effect of AIBA content on swelling time of the St-*g*-PAA/PVA hydrogels. Data are shown as mean ± SD, n = 3.

On the other hand, the AIBA content determines the swelling rate of the hydrogels (Fig. 7). The swelling rate increased significantly with increasing the AIBA content to 0.3 wt% and only 31 s was needed for the swirl to disappear during the swelling rate test, which is attributed to its highly porous structure. With further increasing the AIBA content to 1.1 wt%, the swelling rate gradually decreased and 52 s was needed for the swirl to disappear. Although replacing NaHCO_3_ with AIBA had no obvious influence on the water absorbency, the swelling rate of the hydrogel was significantly enhanced (Figs. 4 and 7), which is in agreement with the morphology mentioned above.

Generally, polymerization of AA is a violent exothermic process resulting in a hydrogel with heterogeneous structure, which inhibits water diffusion into it during water absorption. In this work, AIBA acts as a co-initiator of APS. When the AIBA content is less, a fraction of the produced N_2_ bubbles can take away some water and heat, leading to the polymerization process more steadily. On the other hand, another fraction of the N_2_ bubbles cannot escape from the viscous mixture during the polymerization process, but are trapped in the hydrogel, creating a highly porous structure. As a result, the swelling of the porous hydrogel occurred mainly through capillary action rather than the conventional diffusion, leading to considerable increase in the swelling rate. Moreover, a high porosity means that the internal area of the hydrogel is large. Once water penetrates into the hydrogel via the capillary force, numerous hydrophilic groups are exposed to water molecules, which further accelerates the swelling rate. The gradual decrease of water absorbency and swelling rate when the AIBA content is beyond 0.3 wt% is due to collapse of the three-dimensional network of the hydrogels, which has been confirmed by the SEM micrographs.

### Effects of surface crosslinking on swelling performance of St-*g*-PAA/PVA hydrogels

**Figure 8:**
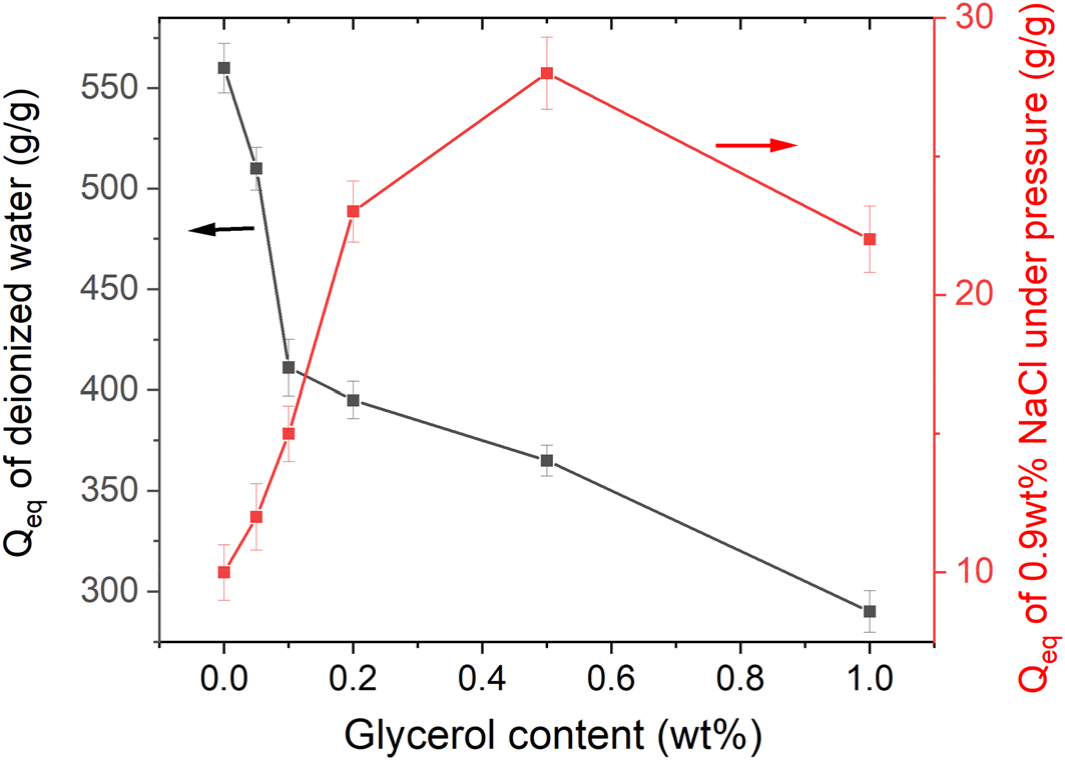
Effect of glycerol content on water absorbency of the St-*g*-PAA/PVA hydrogels in deionized water under normal conditions and in 0.9 wt% NaCl solution under 2 kPa pressure. Data are shown as mean ± SD, n = 3.

**Figure 9:**
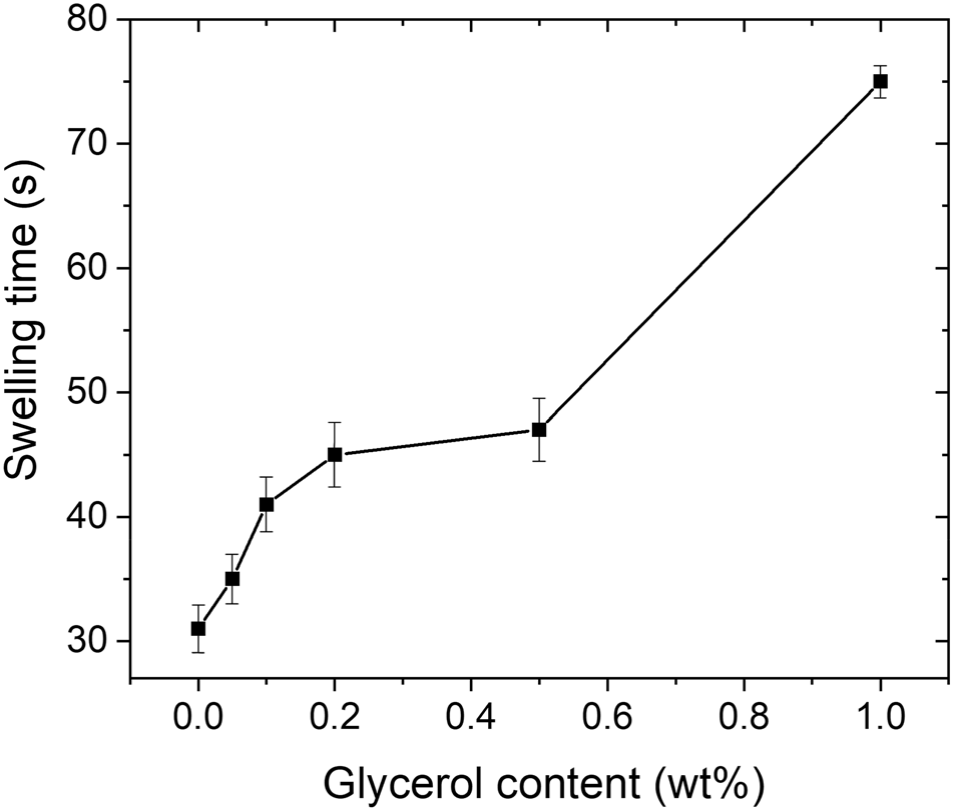
Effect of glycerol content on swelling time of the St-*g*-PAA/PVA hydrogels. Data are shown as mean ± SD, n = 3.

To enhance the water absorbency for saline solution under pressure, the St-*g*-PAA/PVA hydrogel prepared with 0.3 wt% AIBA was further surface crosslinked by glycerol via the esterification reaction. Under high temperature, esterification will happen between the –COOH groups of the hydrogel and –OH groups of glycerol, forming crosslinking points on the surface of the hydrogel. As can be seen from Fig. 8, the water absorbency in deionized water evidently decreased from 560 to 290 g/g with increasing the glycerol content from 0 to 1.0 wt%, which is consistent with the Flory theory about swelling^37^. Meanwhile, the swelling rate decreased with increasing the glycerol content, and a significant increase in the swelling time from 31 to 75 s was necessary for the swirl to disappear in the swelling rate test (Fig. 9). This is because surface crosslinking hinders the capillary penetration of water molecules into the hydrogel network. Note that the content of glycerol should be carefully controlled according to the water absorbency, swelling rate and the water absorbency for saline solution under pressure. Or else, too much glycerol will cause both inside and surface crosslinking reaction of the gel, resulting in serious negative effects.

However, surface crosslinking evidently enhanced the water absorbency in 0.9 wt% NaCl solution under pressure (2 kPa) from 10 g/g (0 wt% glycerol) to 28 g/g (0.5 wt% glycerol), but a further increase in the glycerol content to 1.0 wt% caused evident decrease in the water absorbency under pressure. This is because the surface crosslinking degree increases with increasing the glycerol content, and thus a shell with high crosslinking density was formed on the surface of the hydrogel (Fig. 1). Thus, the absorbed saline water is difficult to be squeezed out under pressure and the water absorbency under pressure is enhanced. However, this is at the scarify of the water absorbency in deionized water and the swelling rate. It is reasonable that both the water absorbency under pressure and the swelling rate decrease when the glycerol content is too high, as the crosslinking degree at the hydrogel surface is too high and the highly crosslinked shell becomes too thick.

## Discussion

In summary, fast-swelling porous St-*g*-PAA/PVA superabsorbent hydrogels with high saline water absorbency under pressure are prepared by the combination of foaming using a new porogen (AIBA) and post surface crosslinking. As proved by experimental results, AIBA is a better porogen than the conventional porogens such as NaHCO_3_ regarding its effects on the porous structure, water absorbency and swelling rate of the hydrogels. In comparison with the hydrogels prepared under the porogen-free condition and using NaHCO_3_ as the porogen, AIBA significantly enhanced the swelling rate of the hydrogels. This is because the decomposition of AIBA and release of N_2_ are more controllable in the whole gelation process. Furthermore, the saline water absorbency was greatly enhanced by post surface crosslinking of the hydrogel with glycerol, but at the sacrifice of the swelling rate. We foresee that the hydrogels may find practical applications in many fields including hygienic products, controlled release and food preservation because of its high saline water absorbency under pressure and fast-swelling behavior. The findings here will shed a new light on the design of fast-swelling hydrogels with high water absorbency under pressure.

## Materials and methods

### Materials

Starch (chemically pure) was obtained from Chengdu Hongbo Industry Co. Ltd., China. PVA (polymerization degree = 1700 ± 50, hydrolysis degree = 99%) was purchased from Lanzhou Chemical Reagent Co. Ltd., China). AA (chemically pure) was purchased from Shanghai Shanpu Chemical Factory, China and was distilled under reduced pressure before use. APS (analytical pure) was purchased from Tianjin Chemical Reagent Factory, China, and was recrystallized before use. MBA (chemically pure) was purchased from Sinopharm Chemical Reagent Co. Ltd, China. AIBA (chemically pure) was purchased from Shanghai Lichen Biological Technology Co. Ltd., China. All solutions were prepared with deionized water.

### Preparation of porous St-g-PAA/PVA hydrogels

Starch (1.0 g) and PVA (1.0 g) were dispersed in 30 mL of deionized water and gelatinized at 90 °C under stirring for 30 min in a 250 mL four-necked flask equipped with a mechanical stirrer, a reflux condenser, a thermometer and a nitrogen line. After being cooled to 40 °C, an aqueous solution (4 mL) containing 49 mg of initiator APS was added. The mixture was stirred for 10 min, and then 17 mL of the solution containing 7.20 g of AA (neutralization degree = 60%) and 12.6 mg of MBA was added. After the mixture was stirred for 10 min, a given amount of AIBA or NaHCO_3_ was added to the flask. Then, the oil bath was slowly heated to 70 °C and kept for 3 h. A nitrogen atmosphere was maintained throughout the reaction period. The obtained St-*g*-PAA/PVA hydrogels were dried in an oven at 70 °C to a constant weight. The dry St-*g*-PAA/PVA gels were ground to a particle size of 40–80 meshes.

The St-*g*-PAA/PVA gel particles were further surface crosslinked via the following procedure. First, a crosslinking solution was prepared by mixing a proper amount of the glycerol crosslinker with 0.05 g of ethanol and 0.30 g of water. Then, the crosslinking solution was sprayed onto 10.0 g of the St-*g*-PAA/PVA gel particles in a flask under vigorous mechanical stirring. After further stirring for 10 min, the crosslinking reaction was carried out by keeping the samples in an oven at 180 °C for 30 min.

### Measurement of equilibrium water absorbency

About 0.05 g of the St-*g*-PAA/PVA gel particles were immersed in 400 mL of deionized water at room temperature for 4 h to reach the swelling equilibrium. Then, the swelling samples were separated from the unabsorbed water using a mesh screen and the hydrogels were allowed to drain on the mesh for 10 min. The water absorbency of the hydrogels was determined by weighing the swelling samples and calculated using the following equation:

1 $$ Q_{{{\text{eq}}}} = \, \left( {m_{{2}} {-}m_{{1}} } \right)/m_{{1}} $$

where *Q*_eq_ (g/g) is the water absorbency calculated as grams of water per gram of the sample. *m*_1_ and *m*_2_ are the weights of the dry sample and swelling sample, respectively. The tests were carried out for three times and the average values were reported.

The water absorbency of the St-*g*-PAA/PVA gel particles in 0.9 wt% NaCl aqueous solution was tested according to the same procedure except that 90 mL of the 0.9 wt% NaCl aqueous solution was used, as the absorbency is low in the saline solution.

### Measurement of swelling rate

50 mL of the 0.9 wt% NaCl aqueous solution was poured into a 100-mL beaker equipped with a 25 mm stirrer. The beaker was placed on an electromagnetic agitator with controllable temperature to ensure the swelling process occurred at 25 °C. Then, 2.0 g of the St-*g*-PAA/PVA gel particles was dispersed into the breaker under stirring at 600 rpm and a swirl was observed on the surface of the NaCl aqueous solution. Meanwhile, the time was recorded until the swirl disappeared. With the swelling of the hydrogel, the viscosity of the mixture increased significantly. Consequently, the swirl became smaller and finally disappeared. The swelling rate of the sample was evaluated by the time that the swirl disappeared. The shorter the time, the faster the swelling of the hydrogel was.

### Measurement of equilibrium water absorbency under pressure

In a glass tube 25 cm in diameter with a lateral porous support, 0.2 g of the St-*g*-PAA/PVA gel particles were spread uniformly on a piece of filter paper. The particles were pressed tightly using a stainless steel column under 2 kPa. Then, the 0.9 wt% NaCl aqueous solution was supplied via a tube to the bottom of the filter paper. After absorption of the saline solution for 4 h by the hydrogel, the weight of the swelling hydrogel was measured. Then, the equilibrium water absorbency under pressure can be calculated according to Eq. (1).

### Morphology observation

The morphology of the St-*g*-PAA/PVA gels was studied using a scanning electron microscope (SEM, JSM-5600LV, JEOL) after coating with a thin layer of gold film.

## Data Availability

The datasets used and/or analysed during the current study available from the corresponding author on reasonable request.
